# Serum Ghrelin, Motilin, and Beta-Endorphin Levels in Infants With Colic and Healthy Infants in Lebanon

**DOI:** 10.7759/cureus.108802

**Published:** 2026-05-13

**Authors:** Reine Elkhawly, Adib Moukarzel

**Affiliations:** 1 Department of Pediatrics, Hôtel-Dieu de France, Beirut, LBN

**Keywords:** colic, ghrelin, lebanon, motilin, β-endorphin

## Abstract

Background: Infantile colic is defined as episodes of fussiness and crying lasting at least three hours per day, three days per week, for more than three weeks. Although its exact cause remains unclear, various biological, gastrointestinal, and psychosocial factors have been proposed. Hormonal alterations, including elevated ghrelin and motilin levels and decreased β-endorphin levels, have been observed in newborns with colic.

Objectives: This study aimed to compare serum levels of ghrelin, motilin, and β-endorphin between colicky and non-colicky infants to better understand the pathophysiology of infantile colic.

Methodology: A total of 68 term infants were included and categorized according to Wessel’s criteria. Blood samples were collected to measure serum levels of ghrelin, β-endorphin, and motilin. The parents completed a questionnaire specifically developed for this study, which collected sociodemographic and anthropometric data, medical history, feeding type, mode of delivery, lifestyle factors, and characteristics of crying episodes. Data analysis was performed using IBM SPSS Statistics for Windows, Version 31.0 (Released 2025; IBM Corp., Armonk, NY, USA).

Results: Infants with colic had higher ghrelin (540.6 ± 436.0 pg/mL versus 478.9 ± 286.2 pg/mL, p = 0.490) and motilin (74.2 ± 33.4 pg/mL versus 61.8 ± 28.3 pg/mL, p = 0.103) levels, and lower β-endorphin levels (1.7 ± 1.7 ng/mL versus 2.1 ± 2.1 ng/ml, p = 0.361) compared to controls, although the differences were not statistically significant. Ghrelin levels were significantly higher in infants meeting all three colic criteria (863.8 ± 473.2 pg/mL) compared to those meeting none (528.7 ± 296.0 pg/mL, p = 0.043) or only one criterion (310.8 ± 117.3 pg/mL, p = 0.028). Beta-endorphin and motilin levels did not differ according to colic severity or feeding type, whereas ghrelin levels were lower in infants receiving mixed feeding compared to those who were formula-fed.

Conclusion: Elevated ghrelin levels in colicky infants, particularly in those meeting all diagnostic criteria, may suggest a possible role of gastric hypermotility in the pathophysiology of colic. The lack of significant changes in β-endorphin levels supports the notion that colic may be a physiological rather than pain-related condition.

## Introduction

Crying serves as a means for infants to express discomfort or needs, particularly during the first few months of life [1]. However, persistent or excessive crying is often referred to as colic. Colic is defined by episodes of fussiness and crying lasting at least three hours per day, three days per week, for more than three weeks, according to Wessel’s criteria [2]. Although colic is a benign and self-limiting condition that typically resolves over time, it may be one of the most challenging conditions experienced during infancy. Colic causes distress and poses a significant burden not only on infants, but also on parents and healthcare providers [3]. Some parents may mistakenly perceive crying as an indication of illness or as a reflection of their caregiving abilities [4]. Parents’ perceptions of the causes of crying, as well as their experiences with the healthcare system, may influence their attitudes toward both their child and the healthcare system even after the crying has subsided [5].

Although the etiology of colic remains unclear, several potential contributing factors have been proposed, including biological, gastrointestinal, and psychosocial factors [1,3]. Colic may involve organic or behavioral mechanisms, including inadequate maternal-infant interaction, maternal anxiety, and difficult infant temperament. Organic hypotheses include food hypersensitivity or allergy, immaturity of gut function, gastrointestinal dysmotility, and pain. In this context, certain hormonal alterations have been associated with colic in newborns, particularly elevated ghrelin and motilin levels [6], along with decreased β-endorphin levels.

Motilin, which is secreted by enteroendocrine cells in the duodenum and jejunum, plays a role in regulating gastric emptying and may also contribute to the mechanisms underlying gastroesophageal reflux [7]. Ghrelin, which is primarily produced in the stomach, is secreted in relation to meal timing and plays a key role in stimulating appetite, gastric motility, and acid secretion [8]. Beta-endorphin is an endogenous opioid neuropeptide synthesized in the hypothalamus and pituitary gland that exerts analgesic effects [9].

This study aimed to evaluate serum levels of ghrelin, motilin, and β-endorphin in infants with colic compared to healthy infants in order to gain insight into the underlying pathophysiology of infantile colic. Motilin and ghrelin were investigated as potential contributors to the gastrointestinal factors associated with colic, while β-endorphin was evaluated to determine whether colic is associated with pain. As secondary objectives, demographic characteristics, household characteristics, and feeding type were also analyzed.

## Materials and methods

Study design and setting

This national observational descriptive study used a cross-sectional design to evaluate serum levels of ghrelin, motilin, and β-endorphin in infants with colic and healthy infants in order to better understand the pathophysiology of infantile colic. The study included infants who were followed at the pediatric department of Hôtel-Dieu de France during routine visits in 2024, including infants who presented with symptoms of colic. The study was approved by the Hôtel-Dieu de France Ethics Committee (CEHDF 381).

Subjects

The study was carried out on 68 term infants aged 1-4 months. Infants were divided into two groups: healthy non-colicky infants (n = 35) and colicky infants (n = 33), who met at least two of the three criteria of Wessel’s definition of colic [2]. Infants were included if they had a gestational age between 37 and 42 weeks, a birth weight appropriate for gestational age (2500-4000 g), and an Apgar score ≥7 at five minutes. Eligible infants had no acute or chronic gastrointestinal diseases, growth-affecting pathologies, or other diseases or abnormalities. In addition, blood samples had to be obtained within fewer than two phlebotomy attempts and within 10 minutes of the first attempt. Complete and differential blood counts were required to fall within normal ranges, and informed parental consent was obtained for all participants. Infants receiving medications or presenting with fever, infections, or other illnesses were excluded from the study.

Recruitment process

After obtaining parental consent and confirming eligibility, parents of eligible infants completed the questionnaire with the assistance of the pediatrician. Venous blood samples were collected between 13:00 and 19:00, at least one hour after feeding. Blood collection was scheduled during the clinic visit and within one hour of questionnaire completion to minimize unnecessary disturbance to the infants.

Data collection

Blood Samples and Biochemical Measurements

Phlebotomy was performed by an experienced registered pediatric nurse in the presence of a pediatrician. Blood was drawn from peripheral veins of the upper or lower limbs into two 2 mL tubes within 10 minutes of the first prick. No more than two attempts were allowed for each infant.

Blood specimens were handled according to the principles of the general protocol for quantitative enzyme immunoassay. Blood was collected into two centrifuge-safe Vacutainer® EDTA plasma tubes and gently inverted several times immediately after collection to ensure anticoagulation. Tubes intended for β-endorphin and motilin measurement were supplemented with aprotinin (0.6 TIU/mL of blood) and gently inverted several times to inhibit protease activity. The blood was then centrifuged at 1,600 × g for 15 minutes at 4°C, after which plasma was collected and stored at -70°C. Tubes intended for ghrelin measurement were supplemented with sufficient 4-(2-aminoethyl)-benzenesulfonyl fluoride (AEBSF) to achieve a final concentration of 1 mg/mL, followed by immediate centrifugation at 2,000-3,000 × g for 15 minutes at -20 ± 5°C. Plasma samples were then acidified with HCl to a final concentration of 0.05 N. Each sample was dated, identified, and stored at -20 ± 5°C. The resulting plasma was subsequently stored at -70°C. Serum levels of ghrelin, β-endorphin, and motilin were measured using specific kits (BA-CEA575HU for motilin, BA-EK02214 for β-endorphin, and DR-4709 for ghrelin) with the Human HumaReader HS ELISA Microplate Reader. A complete blood count and differential count were performed for each infant. Plasma samples were transported on dry ice to the research laboratory of Saint Joseph University, where they were stored at -70°C.

Questionnaire

A questionnaire specifically developed for the objectives of this study (Appendix 1) was completed by the parents during the study visit to collect sociodemographic and anthropometric data, medical history, type of delivery, lifestyle factors, characteristics of crying episodes (duration, age at onset, and reason for crying), and sleeping habits. Crying episodes were evaluated according to Wessel’s criteria, which define colic based on objective reporting data: (1) crying duration greater or less than three hours per day, (2) frequency of occurrence greater or less than 3 days per week, and (3) persistence of crying for more than 3 weeks.

Statistical considerations

Sample Size Calculation

Based on previously reported large effect sizes for serum ghrelin (d ≈ 0.87) and motilin (d > 1.0), 28 subjects per group were required to achieve 90% power at a two-sided α = 0.05. Allowing for attrition and subgroup analyses, 30-35 infants per group were recruited. Accordingly, the study enrolled approximately 60-70 infants in total, including 30-35 colicky infants and 30-35 non-colicky controls, with colicky infants further stratified by feeding type.

Descriptive statistics were performed according to the type of variable (quantitative or qualitative/categorical). Characteristics and outcomes were expressed as mean ± standard deviation, median (minimum-maximum), or counts and percentages with two-sided 95% confidence intervals, as appropriate.

For group comparisons, the type I error rate was set at 5% (two-sided). Depending on data distribution, either Student’s t-test or the Wilcoxon/Mann-Whitney test was used to compare quantitative variables between groups.

Student’s t-test was used to compare colicky and non-colicky infants. One-way analysis of variance (ANOVA) was used for subgroup comparisons.

All analyses were performed using IBM SPSS Statistics for Windows, Version 31.0 (Released 2025; IBM Corp., Armonk, NY, USA). A p-value < 0.05 was considered statistically significant.

## Results

Disposition of infants

A total of 77 infants were enrolled over a nine-month period at Hôtel-Dieu de France. The first subject was enrolled in February 2023, and the last subject in April 2024. Of these, nine did not meet the eligibility criteria for analysis; thus, 68 subjects were included in the final analysis population.

Infant characteristics

Infants had a mean age of 68.6 ± 28.6 days, with a slight predominance of females (46, 57.5%). Most infants were delivered vaginally (41, 60.3%). They lived in relatively crowded households, with family size ranging from three to seven members. Table 1 presents the characteristics of all infants included in the study.

**Table 1:** Infant characteristics

Baseline characteristics	Value (N = 68; 100%)
Age, in days	
Mean ± SD	68.6 ± 28.6
Min-max	30-125
Median	67
Female, n (%)	46 (57.5%)
Weight, in g	
Mean ± SD	5354.3 ± 1118.3
Min-max	3,000-7,500
Median	5,225
Height, in cm	
Mean ± SD	56.7 ± 4.4
Min-max	45-69
Median	56
Type of delivery, n (%)	
Vaginal	41 (60.3%)
C-section	27 (39.7%)
Calculated crowding index	
Mean ± SD	1.1 ± 0.4
Median	1
Feeding mode, n (%)	
Breastfeeding	19 (27.9%)
Formula	18 (26.5%)
Breastfeeding + formula	31 (45.6%)

Colic severity

The three criteria for colic, according to Wessel’s definition, were assessed. Infants met 1.3 ± 1.2 criteria on average. Thirty-five infants (51.5%) presented with no colic symptoms and were classified as non-colicky, while 33 infants (48.5%) met at least two criteria and were classified as colicky. Table 2 presents the proportion of infants experiencing colic symptoms according to Wessel’s definition [2].

**Table 2:** Colic symptoms

Colic criteria	Value (N = 68; 100%)
Number of colic criteria met	
Mean ± SD	1.3 ± 1.2
Min-max	0.0-3.0
Median	1.0
Proportion of infants across number of criteria met, n (%)	
0 criterion	27 (39.7%)
1 criterion	8 (11.8%)
2 criteria	18 (26.5%)
3 criteria (colic infants)	15 (22.1%)
Daily hours of crying categories, n (%)	
<3 hours	53 (77.9%)
≥3 hours	15 (22.2%)
Crying more than three days per week, n (%)	36 (52.9%)
Crying more than three weeks, n (%)	38 (55.9%)

Hormone levels in non-colicky versus colicky infants

Ghrelin, β-endorphin, and motilin levels were measured in infants’ blood samples. Infants with colic showed higher ghrelin levels (540.6 ± 436.0 pg/mL versus 478.9 ± 286.2 pg/mL, p = 0.490), lower β-endorphin levels (1.7 ± 1.7 ng/mL versus 2.1 ± 2.1 ng/mL, p = 0.361), and higher motilin levels (74.2 ± 33.4 pg/mL versus 61.8 ± 28.3 pg/mL, p = 0.103) compared to infants without colic (Table 3).

**Table 3:** Hormone levels in colicky and non-colicky infants t-tests were performed to compare the non-colic and colic groups.

Hormones	Infants (N = 68; 100%)		
Ghrelin, in pg/mL	Non-colicky	Colicky	Total
Mean ± SD	478.9 ± 286.2	540.6 ± 436.0	508.9 ± 365.1
Min-max	16.2-1,062.6	170.1-1,861. 0	117.1-1,163.5
Median	380.0	324.0	357.1
	p = 0.490		
Beta-endorphin, in ng/mL	Non-colicky	Colicky	Total
Mean ± SD	2.2 ± 2.0	1.7 ± 1.7	2.0 ± 1.9
Min-max	0.6-7.8	0.5-3.8	0.5-7.8
Median	1.0	1.0	1.0
	p = 0.361		
Motilin, in pg/mL	Non-colicky	Colicky	Total
Mean ± SD	61.8 ± 28.3	74.2 ± 33.4	67.8 ± 31.3
Min-max	13.0-170.2	24.8-176.0	13.0-170.2
Median	59.2	64.5	63.4
	p = 0.103		

Variation in serum hormone levels according to colic severity and feeding mode

Serum hormone levels were analyzed according to the number of colic criteria met and type of feeding (Table 4). While serum β-endorphin (p = 0.761) and motilin (p = 0.320) levels did not differ significantly across groups, serum ghrelin levels were significantly higher in infants meeting all three colic criteria (863.8 ± 473.2 pg/mL) compared to those meeting none (528.7 ± 296.0 pg/mL, p = 0.043) or only one criterion (310.8 ± 117.3 pg/mL, p = 0.028). Serum β-endorphin and motilin levels did not vary according to feeding type. However, serum ghrelin levels were significantly higher in formula-fed infants compared to those receiving mixed feeding (695.3 ± 520.6 pg/mL versus 403.0 ± 249.5 pg/mL, p = 0.032).

**Table 4:** Variation in serum hormone levels according to colic severity and feeding mode ANOVA test was performed to compare between groups.

Colic criteria/feeding mode	Hormone level (N = 68; 100%)		
	Ghrelin, in pg/mL	Beta-endorphin, in ng/mL	Motilin, in pg/mL
Number of met colic criteria			
0 criteria, N = 27	528.7 ± 295.6	2.2 ± 2.1	61.2 ± 30.7
1 criteria, N = 8	310.8 ± 117.3	2.3 ± 2.0	63.8 ± 19.5
2 criteria, N = 18	271.3 ± 66.5	1.7 ± 1.9	69.7 ± 24.5
3 criteria, N = 15	863.8 ± 473.2	1.8 ± 1.5	79.6 ± 42.0
Feeding mode			
Breastfeeding, N = 19	504.9 ± 287.2	1.2 ± 0.9	53.9 ± 20.9
Formula, N = 18	695.3 ± 520.6	2.7 ± 2.6	74.4 ± 34.8
Breastfeeding + formula, N = 31	403.0 ± 249.5	1.9 ± 1.8	72.5 ± 32.6

## Discussion

This observational cross-sectional study aimed to investigate the potential role of ghrelin, motilin, and β-endorphin serum levels in the pathophysiology of infantile colic by comparing hormone levels between colicky and non-colicky infants treated at Hôtel-Dieu de France. Although the differences in hormone levels between the two groups did not reach statistical significance, several trends were observed that may contribute to understanding the biological mechanisms underlying infantile colic.

In this study, infants with colic exhibited higher serum ghrelin levels compared to non-colicky infants, suggesting a potential association between elevated ghrelin levels and colic symptoms. Ghrelin is known to stimulate appetite and enhance gastrointestinal motility and acid secretion, and increased ghrelin levels may promote gastrointestinal hyperactivity, a mechanism that may contribute to abdominal discomfort and excessive crying in infants with colic [10].

Interestingly, subgroup analysis revealed significantly higher ghrelin levels among infants who fulfilled all three of Wessel’s criteria compared to those meeting none or only one criterion. This finding strengthens the hypothesis that ghrelin may play a role in the severity or presence of colic symptoms [6].

While motilin levels were also higher in colicky infants, the difference was not statistically significant. Given motilin’s role in regulating gastric emptying and intestinal motility, elevated levels may reflect altered gastrointestinal dynamics, leading to increased intestinal contractility and accelerated transit time, which may contribute to the intestinal discomfort experienced during colic episodes [6]. However, the current data do not confirm a definitive association.

The concurrent elevation of motilin and ghrelin levels in colicky infants is consistent with findings reported by Savino et al., who previously observed similar results [6].

In contrast, β-endorphin levels were lower in colicky infants, although the difference was not statistically significant. This finding suggests that colic may not be a pain-driven condition requiring an analgesic response, supporting theories that colic is a physiological rather than pathological phenomenon.

Gastrointestinal physiology, microbial composition, and hormonal regulation may also be influenced by early nutritional patterns [10,11].

Feeding practices did not significantly influence motilin or β-endorphin levels; however, ghrelin levels were lower in mixed-fed infants compared to formula-fed infants. This may indicate a complex interaction between feeding patterns and gastrointestinal hormone regulation in infants with colic, further emphasizing the potential benefits of breastfeeding.

Overall, although statistically significant differences were not consistently identified, the hormonal trends observed in this study suggest that ghrelin and motilin may play a role in the complex pathophysiology of infantile colic. Current evidence increasingly supports a multifactorial model involving gastrointestinal immaturity, dysregulated gut motility, alterations in the microbiota, and gut-brain axis interactions.

This study has several limitations. The monocentric cross-sectional design may limit the generalizability of the results and does not allow causal inference. Potential confounding factors, such as feeding type and maternal factors, were not fully controlled. In addition, the high variability in measurements, reflected by the large standard deviations, may have affected the precision and reliability of the observed associations.

## Conclusions

Higher ghrelin levels in colicky infants may suggest a potential role in gastrointestinal hypermotility and functional gastrointestinal disturbances. In contrast, unchanged β-endorphin levels may indicate that pain-related mechanisms are not prominently involved. Further studies with larger sample sizes and longitudinal designs are needed to confirm these findings and explore their clinical significance.

## Appendices

Appendix 1. Questionnaire

رمز الطفل: Child code:

عمر الطفل بالأشهر: Child’s age (in months):

هل يعاني الطفل من نوبات من البكاء الشديد لمدة ثلاث ساعات يومياً؟ Does the child have episodes of intense crying for three hours per day?

☐ نعم (Yes) ☐ كلا (No)

هل يحدث ذلك لأكثر من ثلاثة أيام في الأسبوع؟ Does this occur for more than three days per week?

☐ نعم (Yes) ☐ كلا (No)

هل يستمر ذلك لأكثر من ثلاثة أسابيع متتالية؟ Does this continue for more than three consecutive weeks?

☐ نعم (Yes) ☐ كلا (No)

في أي وقت من النهار يكون بكاء الطفل أكثر؟ At what time of the day is the child’s crying most frequent?

☐ في الصباح - Morning ☐ خلال النهار - During the day ☐ في المساء - Evening ☐ خلال الليل - Night

هل يعاني الطفل من مشاكل صحية معينة؟ Does the child have any specific health problems?

☐ كلا (No) ☐ نعم (Yes) - حدد / Specify:

هل يوجد سبب معين يدفع الطفل إلى البكاء؟ Is there a specific reason that makes the child cry?

هل يشد الطفل جسمه ويسحب رجليه إلى الأمام ويمرر غازاً أثناء البكاء؟ Does the child tense their body, pull their legs forward, and pass gas while crying?

☐ دائماً - Always ☐ أحياناً - Sometimes ☐ نادراً - Rarely

هل يسبب حليب البقر أو أي طعام آخر حساسية للطفل؟ Does cow’s milk or any other food cause the child an allergy?

كم كان وزن الطفل عند الولادة؟ What was the child’s weight at birth?

هل تساعد الطفل على التجشؤ بعد تناوله الطعام؟ Do you help the child burp after feeding?

☐ نعم (Yes) ☐ كلا (No)

هل يتعرض الطفل لدخان السجائر؟ Is the child exposed to cigarette smoke?

☐ نعم (Yes) ☐ كلا (No)

كم شخصاً يسكن في المنزل؟ How many people live in the house?

من كم غرفة يتألف المنزل؟ How many rooms does the house have?
